# Multi-level assessment of obsessive-compulsive disorder (OCD) reveals relations between neural and neurochemical levels

**DOI:** 10.1186/s12888-020-02913-5

**Published:** 2020-11-25

**Authors:** Kathrin Viol, Günter Schiepek, Martin Kronbichler, Arnulf Hartl, Carina Grafetstätter, Peter Strasser, Anna Kastinger, Helmut Schöller, Eva-Maria Reiter, Sarah Said-Yürekli, Lisa Kronbichler, Brigitte Kravanja-Spannberger, Barbara Stöger-Schmidinger, Marc-Thorsten Hütt, Wolfgang Aichhorn, Benjamin Aas

**Affiliations:** 1 Institute of Synergetics and Psychotherapy Research, Paracelsus Medical University, Ignaz-Harrer-Strasse 79, 5020 Salzburg, Austria; 2 Department of Psychosomatics and Inpatient Psychotherapy, University Hospital for Psychiatry, Psychotherapy and Psychosomatics, Paracelsus Medical University, Salzburg, Austria; 3 Department of Psychology, Ludwig Maximilians University, Munich, Germany; 4 Centre for Cognitive Neuroscience and Department of Psychology, Paris Lodron University of Salzburg, Salzburg, Austria; 5 Neuroscience Institute, Christian-Doppler Medical Center, Paracelsus Medical University, Salzburg, Austria; 6 Institute for Ecomedicine, Christian-Doppler Medical Center, Paracelsus Medical University, Salzburg, Austria; 7 Institute of Biochemical Diagnostic, Paracelsus Medical University, Salzburg, Austria; 8 Department for Radiotherapy and Radio-Oncology, Christian-Doppler University Hospital of the Paracelsus Medical University, Salzburg, Austria; 9 Department for Neurology, Christian-Doppler University Hospital of the Paracelsus Medical University, Salzburg, Austria; 10 Department of Life Sciences and Chemistry, Jacobs University, Bremen, Germany; 11 Department of Child and Adolescent Psychiatry, Psychosomatics and Psychotherapy, University Hospital, Ludwig Maximilians University, Munich, Germany; 12 Faculty of Psychology and Educational Sciences, LMU Munich, Munich, Germany

**Keywords:** OCD, fMRI, Psychotherapy process, Treatment outcome, Neurochemistry, Multi-level, Dopamine, Cortisol, IL-6

## Abstract

**Background:**

While considerable progress has been made in exploring the psychological, the neural, and the neurochemical dimensions of OCD separately, their interplay is still an open question, especially their changes during psychotherapy.

**Methods:**

Seventeen patients were assessed at these three levels by psychological questionnaires, fMRI, and venipuncture before and after inpatient psychotherapy. Seventeen controls were scanned at comparable time intervals. First, pre/post treatment changes were investigated for all three levels separately: symptom severity, whole-brain and regional activity, and the concentrations of cortisol, serotonin, dopamine, brain-derived neurotrophic factor (BDNF), and immunological parameters (IL-6, IL-10, TNFα). Second, stepwise linear modeling was used to find relations between the variables of the levels.

**Results:**

The obsessive-compulsive, depressive, and overall symptom severity was significantly reduced after psychotherapy. At the neural level, the activity in the anterior cingulate cortex (ACC), in frontal regions, in the precuneus, and in the putamen had significantly decreased. No significant changes were found on the neurochemical level. When connecting the levels, a highly significant model was found that explains the decrease in neural activity of the putamen by increases of the concentrations of cortisol, IL-6, and dopamine.

**Conclusion:**

Multivariate approaches offer insight on the influences that the different levels of the psychiatric disorder OCD have on each other. More research and adapted models are needed.

**Supplementary information:**

The online version contains supplementary material available at 10.1186/s12888-020-02913-5.

## Background

### Multivariate approach to OCD

Psychiatric disorders are complex phenomena that comprise multiple variables from different levels, ranging from neural activity, neurochemistry, and genes, to a variety of psychological, social, and environmental factors. These variables do, most likely, not act independently, but are interlinked and influence each other. This might not only be true for variables within a level, but also between levels, where processes at one scale may cause or shape the processes on other scales [1]. Hence, correlations between two variables do not seem to be sufficient when trying to explain psychiatric disorders considering the multivariate nature of the biopsychosocial system [2]. While the availability of big data and open access datasets has led to first attempts on multilevel research in recent years, several important limitations have recently been identified [1]. Next to the criticism that the same open-access datasets are used repeatedly, the authors stress that longitudinal designs would be important in order to elicit underlying mechanisms of psychiatric disorders and change processes. Moreover, most studies focus on connecting the different scales of the brain (micro, meso, and macro scale), but do not take into account the well-known interactions with the neurochemical and the psychological level.

In this study, individuals with obsessive-compulsive disorder (OCD) were assessed on three different levels before and after psychotherapy: the psychological, the neural, and the neurochemical level. Psychotherapeutic treatments can effectively reduce the symptoms of patients [3] and induce changes both at the neural and chemical level [4, 5]. By conceptualizing the treatment as an experimental manipulation to learn how plasticity in the brains of patients relates to changes in symptoms, we account for the shortfall of previous studies. Rather than determining the effectiveness of psychotherapy, the aim of this study is to investigate the manifestation of psychotherapeutic changes on all three levels, and to generate data-based models of their interplay.

### Current models of OCD

Models of psychiatric disorders like OCD usually focus on one level only. For OCD, such models are available for the psychological level, the neural level, and the neurochemical level. The definition of OCD is based on cognitive-emotional-behavioral aspects, i.e., the psychological level. The DSM-5 characterizes the illness by the persistent intrusion of unwanted thoughts or imaginations (obsessions) and/or the urge for repetitive, ritualistic behaviors or mental acts (compulsions) [6]. The behavior of patients is based on maladaptive cognitive processes and believes, e.g., inflated personal responsibility, the overestimation of threat, perfectionism, and the intolerance of uncertainty (cognitive model of OCD) [7]. These impairments have been associated with differences on the neural as well as on the biochemical levels in OCD patients compared to healthy controls, suggesting these alterations to underlie the illness. The predominant model of the neural level suggests that OCD results from impairments within the cortico-striato-thalamic-cortical circuit (CSTC) [8–10]. This circuit includes an affective loop, which comprises the ventral anterior cingulate cortex (ACC), anterior/lateral orbitofrontal cortex (OFC), parts of the basal ganglia (putamen, nucleus caudatus, pallidum), the medio-dorsal part of the thalamus, the hippocampus, and the amygdala. The other part of the CSTC-network is the cognitive loop, which comprises the ventro- and dorsolateral prefrontal cortex (vlPFC/dlPFC), dorsal ACC, posterior OFC, posterior parietal cortical regions, parts of the basal ganglia (putamen, N. caudatus, globus pallidus), and the ventro-anterior part of the thalamus [8, 11, 12]. These circuits may, however, not be exhaustive, and additional regions and (sub-) circuits have been proposed to play a role, too [10, 13]. Consistently, these OCD-related regions are hyper-activated in patients compared to controls [14].

Several studies assessed the effects of psychological interventions (mostly cognitive-behavioral therapy) on the neural activity (Table 1) using functional magnetic resonance imaging (fMRI). Comparable to the findings of neural correlates in the cross-sectional studies, the brain regions that were subject to change included core regions of the CSTC-circuit, but were not limited to those (Table 1).

**Table 1:** Literature review of brain regions with significant change in neural activity for obsessive-compulsive disorder (OCD) patients before and after psychotherapy. Please note that this list only includes task-related fMRI studies

Brain region	Change during psychotherapy ↑ increase or ↓ decrease	
	executive tasks	symptom provocation
amygdala		↑ Olatunji et al. [15]
anterior cingulate cortex	↑ Huyser et al. [16] ↑ Verfaillie et al. [17]	↓ Morgiève et al. [18] ↓ Schiepek et al. [13] ↓ Nakao et al. [19]
anterior temporal pole		↑ Olatunji et al. [15]
cerebellum	↑ Nakao et al. [19] ↑ Nabeyama et al. [20]	↓ Nakao et al. [19]
cuneus		↓ Schiepek et al. [13]
fusiform gyrus	↓ Nabeyama et al. [20]	
hippocampus	↓ Nakao et al. [19]	
insula	↓ Lázaro et al. [21] ↓ van der Straten et al. [22]	↓ Schiepek et al. [13]
middle cingulate cortex	↓ Nakao et al. [19]	
middle frontal cortex	↓ Nabeyama et al. [20] ↓ Nakao et al. [19]	
nucleus accumbens		↓ Baioui et al. [23]
nucleus caudatus	↑ Freyer et al. [24] ↑ Verfaillie et al. [17]	↓ Baioui et al. [23]
occipital cortex	↓ Nakao et al. [19]	↓ Nakao et al. [19]
orbitofrontal cortex	↓ Nabeyama et al. [20]	↓ Baioui et al. [23] ↓ Morgiève et al. [18] ↓ Nakao et al. [19]
parahippocampus	↓ Nabeyama et al. [20]	
parietal cortex	↑ Nakao et al. [19]	↓ Schiepek et al. [13]
precuneus	↓ Nabeyama et al. [20]^a^ ↑ Nabeyama et al. [20]^b^	
prefrontal cortex	↑ Huyser et al. [16] ↑ Nakao et al. [19]	↓ Baioui et al. [23] ↓ Schiepek et al. [13]
premotor region	↑ Huyser et al. [16]	
putamen	↓ Freyer et al. [24] ↓ Lázaro et al. [21] ↑ Nakao et al. [19] ↑ Verfaillie et al. [17]	↓ Nakao et al. [19]
supramarginal gyrus		↓ Baioui et al. [23]
temporal cortex (middle and superior)	↑ Nakao et al. [19]^a^ ↓ Nakao et al. [19]^b^	↓ Nakao et al. [19]
thalamus		↓ Nakao et al. [19]

^a^for left hemisphere

^b^for right hemisphere. Note that there were no exclusion criteria, so the table above includes also studies without controls, and results without correction for multiple comparisons

Several models have been proposed that aim to explain the OCD pathology on the neurochemical level. Most prominent is the dopamine-serotonin hypothesis [12, 25], but there are also hints that immunological aspects may play a role [26], e.g., the concentrations of the proteins interleukin 6 and 10 (IL-6/IL-10), or the tumor necrosis factor (TNF-α), that are involved in the regulation of the immune response. In addition, stress has commonly been associated with the development of psychiatric disorders [27]. We therefore investigated the concentrations of cortisol and the brain-derived neurotrophic factor (BDNF). The latter is also claimed to be relevant for a successful therapy [28, 29], since it is involved in various neural functions such as axon growth, dendrite pruning, and the expression of proteins [30]. Most importantly, BDNF has been shown to interact with neurotransmitters and might therefore play an important role in a multivariate model.

### Aims and hypotheses

**Fig. 1:**
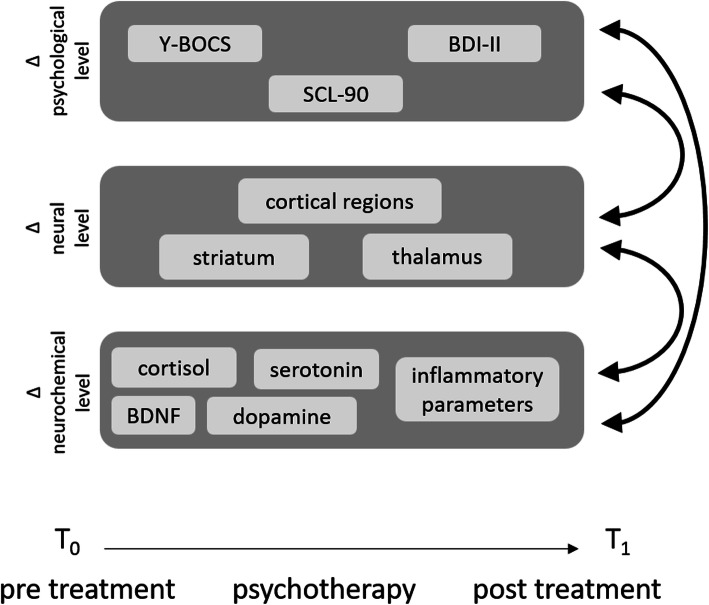
Study design of the multi-level assessment of patients with obsessive-compulsive disorder (OCD) before and after psychotherapy. The psychological level comprises the OCD symptom severity (Y-BOCS), depression symptom severity (BDI-II) and overall symptom severity (SCL-90). The neural level consists of the regions of the cortico-striato-thalamic-cortical network (CSTC). On the neurochemical level, the concentrations of the neurotransmitters dopamine and serotonin, inflammatory parameters (IL-6, IL-10, and TNF-α), cortisol, and the brain-derived neurotrophic factor (BDNF) were assessed. For all variables, the pre-post difference (Δ) was calculated and evaluated

The numerous variables on different levels that have been associated with OCD ask for a multivariate systemic approach to OCD. The levels and variables investigated in our study are depicted in Fig. 1. The choice of variables for the psychological level was based on the symptoms of the patients, which define the illness according to DSM-5. The Yale-Brown Obsessive-Compulsive Scale (Y-BOCS) [31] assesses these symptoms on the behavioral, emotional, and cognitive level and is the most commonly used questionnaire in OCD research [32]. Depression is the most common comorbidity of OCD, and a considerable overlap in factors associated with OCD as well as Major Depressive Disorder has been observed both on the neural and the neurochemical level [14, 33, 34]. Depressive symptom severity was assessed by the Beck Depression Inventory II (BDI-II) [35]. In order to account for other comorbidities, the overall severity of symptoms was evaluated by the Symptom Checklist-90-R (SCL-90) [36]. The hypotheses consisted of a reduction of the three symptom scores.

At the neural level, a reduction of the activity of cortical regions (ACC, OFC, PFC), striatal regions (putamen, n. caudatus, n. accumbens), and the thalamus, which comprise the CSTC-network, was expected after treatment.

At the neurochemical level, a reduction of the concentration of cortisol and of the immune parameters was predicted, as well as an increase in BDNF, serotonin and dopamine.

Exploratory stepwise linear regression was used to identify models that can explain the change at one level by the changes at another level, i.e., that can explain the change in symptom severity by changes in neural activity, and a model than can explain the changes in neural activity by changes in the concentrations of neurochemical parameters.

## Methods

### Study procedure and participants

Within the first and the last week of admission to inpatient or day-patient treatment, patients underwent a psychological assessment, an fMRI scan, and a venipuncture. The same procedure (but without the venipuncture) was applied to the controls matched by age and gender at comparable time intervals. The average interval between the two time points was 86 days (*SD* = 25) for patients and 84 days (*SD* = 33) for controls. Note that, although the study aims (also) to investigate correlates of therapeutic change, 17 healthy participants were chosen as a control group representing “no change”, since (1) we were also interested in mechanisms of the OCD pathology, which requires healthy controls, and (2) patients on waiting list have repeatedly been questioned as an appropriate control group (for an overview see [37]).

The patient sample consisted of 17 inpatients (6 men and 11 women, mean age 43.5 years, *SD* = 1.7), receiving psychotherapy at the Department of Inpatient Psychotherapy, University Hospital of Psychiatry, Psychotherapy and Psychosomatics of the Paracelsus Medical University, Salzburg, Austria. Psychotherapeutic treatment consisted of an integrative approach including weekly individualized psychotherapy sessions based on the concept of cognitive-behavioral therapy with an experienced therapist, psychoeducation, mentalization/mindfulness training, focused groups, skills training following Dialectic Behavioral Therapy, music and art therapy, indoor climbing, and walking.

Patients were eligible to participate in the study if obsessive-compulsive disorder was the main illness by clinical judgement based on ICD-10 and DSM-IV criteria and on the Structured Clinical Interview for DSM-IV Axis I disorders (SCID-I) [38]. Exclusion criteria consisted of neurological impairment and/or neurological diseases, acute psychosis, substance abuse, and/or suicidality. The mean score of the Yale-Brown Obsessive Compulsive Scale (Y-BOCS) was 26.7 (*SD* = 8.8), which ranks the sample on the medium to upper end of symptom severity. Comorbidities, as commonly found in OCD patients, included depression (8 patients), social phobia (2 patients, in addition to depression) and one each from the schizophrenic spectrum, alcohol and substance abuse (currently abstinent), and posttraumatic stress disorder. The mean depression score (BDI-II) was 29.0 (*SD* = 9.4) for patients and 1.2 (*SD* = 1.5, *p* < .001) for controls. All but one patient took some kind of antidepressant (mostly SSRI), 7 of them in addition neuroleptics, 3 anticonvulsants, 2 benzodiazepine and 1 lithium. One patient also had to be medicated for high blood pressure, thyroid dysfunction, and incontinence.

The study was approved by the Ethics Commission Salzburg (Ethikkommission Land Salzburg, No. 415-E/1203/5–2012). Detailed information on the study was provided and written informed consent was obtained from all participants according to the Declaration of Helsinki.

### Psychological variables

At the psychological level, the overall symptom severity was assessed by the Global Severity Index of the Symptom Checklist-90-R (SCL-90) [36, 39]; depressive symptoms were assessed by the Beck Depression Inventory II (BDI-II) [35, 40], and the obsessive-compulsive symptom severity by the Yale-Brown Obsessive-Compulsive Scale (Y-BOCS) [31, 41].

### Neurochemical variables

On days with a scheduled fMRI scan, blood was drawn from patients by venipuncture at 8 a.m. on an empty stomach. The samples were centrifuged for 10 min at 3000 rpm and stored at − 80 °C until analyzed at the Institute for Ecomedicine of the Paracelsus Medical University, Salzburg, Austria. From the serum, the concentration of the following 7 parameters was extracted: cortisol, brain-derived neurotrophic factor (BDNF), interleukin 6 (IL-6) and 10 (IL-10), tumor necrosis factor α (TNFα), dopamine and serotonin. For serotonin and dopamine, the analysis kits ELISA (Labor Diagnostika Nord) were used, and Human 5Plex Analytes (Thermo Fisher Scientific) for BDNF, Cortisol, IL-6, IL-10, and TNFα.

### Neural variables (fMRI)

Functional and structural fMRI images were acquired with a 3 T Siemens TIM TRIO whole-body scanner. State-of-the art preprocessing was performed using an adaption of the Statistical Parametric Mapping software package SPM12 (Wellcome Department of Cognitive Neurology, London) implemented in Matlab (Mathworks, release 13a) including realignment, despiking, correction of distortions using the fieldmap of each participant, slice time correction, normalization to MNI-space and smoothing with a 6 mm FWHM Gaussian kernel. For details on the process of acquisition and preprocessing see supplement A. For symptom provocation during the fMRI scan, pictures from 4 different categories were shown to patients and controls: individual OCD-provoking photos, standardized OCD-provoking photos from the Maudsley Obsessive-Compulsive Stimulus Set [42], and disgusting and neutral pictures from the International Affective Pictures Set [43]. 40 pictures from each category were displayed in a pseudo-randomized order with a duration of 4 s per picture. The details of the acquisition and selection process of the individual pictures, which were taken in the domestic environment of the patients, can be found in Viol et al. [44].

The difference between individual OCD and neutral pictures was used to assess the change in neural activity before and after psychotherapy for patients vs. controls. A whole-brain analysis was performed with the Multivariate and Repeated Measures (MRM)-Toolbox^1^ for SPM. An ANOVA was set up with 1 between-subject factor (patients/controls) and two within-subject factors (pre/post and OCD/neutral stimuli). Whole brain analysis was calculated on cluster level with a threshold of *p* < .05 (FWE-corrected, based on *p* < .001) and the permutation approach with 5000 permutations.

In addition, a region-of-interest (ROI) analysis was performed, assessing only regions of the brain that have previously been shown to have changed during psychotherapy in OCD patients. ROI analyses of specific pre-defined regions are commonly used in fMRI studies aiming to investigate pre-post treatment differences, since one usually does not assume that the neural activity in the whole brain has changed during psychotherapy, but only in regions specific to the illness. Correction for multiple comparisons in whole-brain analyses might therefore lead to false negative results. To avoid circular analysis [45], we did not choose regions based on our sample, but the regions described in the literature (Supplement B/Table S1). With the MarsBaR toolbox^2^ [46], ROIs were defined with a radius of 10 mm around each of these 32 voxels. The mean activity for the contrast “individual OCD vs. neutral pictures” within each ROI was extracted for each scan and subject. These mean activities (β-values) were then entered into a 2 × 2 repeated measures ANOVA in Matlab with one within-subject factor (time) and one between-subject factor (group). Correction for multiple comparisons was calculated with a Matlab implementation of the R-function *p.adjust.*^3^ As method, the false-discovery rate (FDR) algorithm by Benjamini and Hochberg [47] was used. The effect sizes η^2^ were calculated with the Matlab toolbox *MES* for calculating effect sizes in neuroscience [48, 49].

### Regression model

In order to find relations between the psychological, neurochemical and neural level, stepwise linear regression in MATLAB was used. As dependent variables, we used those variables from the psychological and from the neural level that had shown a significant difference on the group level. The variables of the lower level were the possible predictors. In stepwise linear modeling, the algorithm starts with a constant model and successively adds or removes variables (and all possible interaction terms) one step at a time until no more can be added or removed according to the criterion, here the maximization of the Akaike information criterion (AIC).

The *p*-values of the predictor variables were then corrected for multiple comparisons (false discovery rate algorithm, FDR) and all variables that were significantly different from zero (*p* < .05, FDR-corrected) were entered into a conventional linear regression model. The robustness of the fit was evaluated by the option ‘RobustOpts’,‘on’ in function *fitlm* in MATLAB (supplement D).

## Results

Note that all changes during psychotherapy were calculated pre- minus post-values, thus a positive Δ-value is equivalent to a decrease (pre > post), and a negative value to an increase.

### Psychological outcome

The patients’ symptoms were significantly reduced at the end of therapy for all outcome measures. The mean Y-BOCS score was reduced by 9 points (30%, *SD* = 9, *p* < .01). Depression improved even more, with a reduction of the mean BDI-II scores by 11 points (47%, *SD* = 9, *p* < .01). Also, the average overall symptom severity assessed by the SCL-90-R GSI scale was reduced by .53 points (29%, *SD* = .61, *p* = .01). Three patients did not fill in the BDI-II and SCL-90-R at the last scan.

### Immunological and endocrinal parameters

When comparing the pre- and post-treatment concentration of the blood parameters of the patients, no significant differences were detected (Supplement C). Still, the mean values for BDNF, dopamine and serotonin shifted in the expected directions (increase). For cortisol, however, the opposite of the expected decrease was measured at the end of the therapy.

### Neural activity

**Fig. 2:**
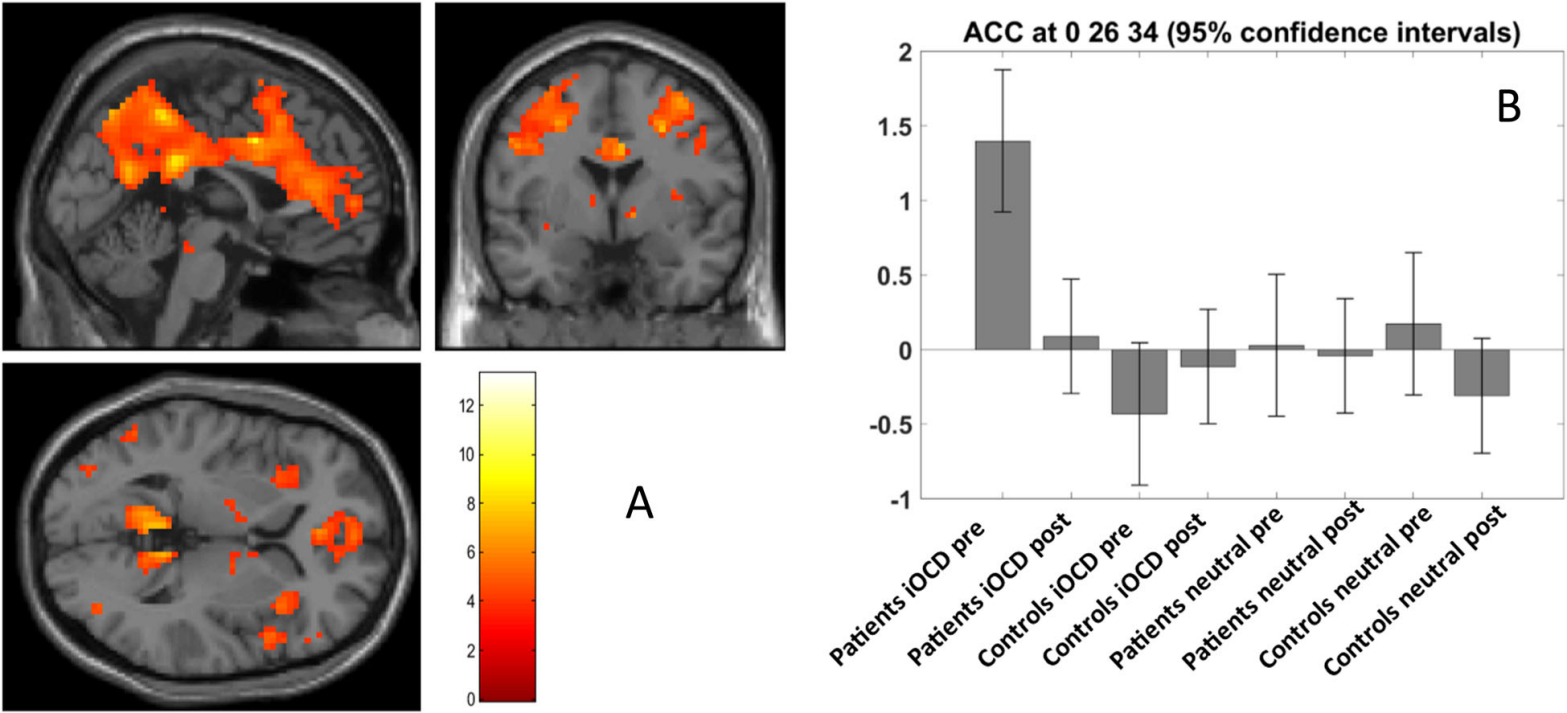
Differences in neural activity before and after psychotherapy. **a**: Decreased activity of patients compared to controls for individual OCD > neutral pictures during treatment, *p* < 0.001 uncorrected for visualization [44]. **b**: Contrast estimates of the voxel at x = 0, y = 26, z = 34, which is part of the cluster whose neural activity was found to be significantly altered in the ANOVA for the group x time x condition interaction term, *p* < .05 FDR-corrected

At the whole-brain level, the 3 × 2 ANOVA revealed a change in brain activity in the anterior cingulate cortex (ACC) for the *condition x group x time* interaction term. The three peaks within the cluster are given in Table 2. Figure 2a shows the neural activity for patients > controls for the contrast “individual OCD vs. neutral pictures” that has changed during psychotherapy. The contrast estimates (Fig. 2b) at x = 0, y = 26, z = 34 show a clear hyperactivity in the ACC in patients at the beginning of the psychotherapy compared to controls, which is reduced after treatment. The contrast estimates of the other two peaks are equivalent (not shown).

**Table 2:** Peaks of neural activity within the cluster and family wise error (FWE)-corrected *p-*values for the *group x time x condition* interaction term of the 2 × 3 ANOVA on whole-brain level

Region	L/R	coordinates			cluster	extent	*p* (FWE)
		x	y	z			
ACC	–	0	26	34	1	52	.045
	R	6	29	25			
	L	-3	17	37			

*ACC* anterior cingulate cortex, *L* left hemisphere, *R* right hemisphere

In addition, the region-based approach (ROI analysis) revealed further regions with significant changes in patients (compared to controls) before and after psychotherapy (*time x group* interaction term of the 2 × 2 repeated measures ANOVA; here the condition already consisted of the difference between individual OCD (iOCD) and neutral pictures). Table 3 shows the results for the regions with significant pre-post differences after correction for multiple comparisons; the results of the other regions can be found in Supplement B, Table S1.

**Table 3:** Group mean (*SD*) values of the neural activity (β estimates) and false discovery rate (FDR)-corrected *p-*values for the *group x time* interaction term of the repeated measures ANOVA. Also given are the effect sizes *η*^2^, which are all in the medium range [50]. Only the significant regions are shown (see Supplement B, Table S1 for the whole list)

Region	coordinates				OCD patients		healthy controls		*p* (FDR)	*η*^*2*^
		x	y	z	pre	post	pre	post		
ACC	L	-9	21	42	.81 (.63)	.24 (.64)	−.26 (.34)	.00 (.47)	.03	.07
	L	−4	28	24	.48 (.41)	.19 (.40)	−.13 (.25)	.06 (.24)	.02	.06
OFC	L	−45	17	−8	.80 (.64)	.27 (.80)	−.22 (.46)	.02 (.62)	.03	.10
	L	−48	17	−5	.73 (.64)	.23 (.86)	−.26 (.47)	−.02 (.64)	.03	.09
PCu	R	4	−72	46	1.86 (1.22)	.54 (1.22)	.35 (.87)	.29 (.69)	.03	.07
Putamen	L	−15	2	1	.27 (.40)	−.03 (.31)	−.01 (.26)	.01 (.21)	.03	.06

*ACC* anterior cingulate cortex, *OFC* orbitofrontal cortex, *PCu* precuneus, *L* left hemisphere, *R* right hemisphere. Values of *η*^*2*^ between 0.6 and 0.13 are considered medium effect sizes

### Relations between the levels

The aim of the second part of the paper was to find relations in the changes between levels. To facilitate reading, “changes in” will be denoted by Δ in the following sections.

The Δ activity in the putamen was explained by the changed concentration of cortisol, interleukin 6, and dopamine:

$$ -\Delta putamen=.0002\cdot \Delta cortisol+.0166\cdot \Delta IL6+.0024\cdot \Delta dopamine+\mathrm{0.2021.} $$

**Fig. 3:**
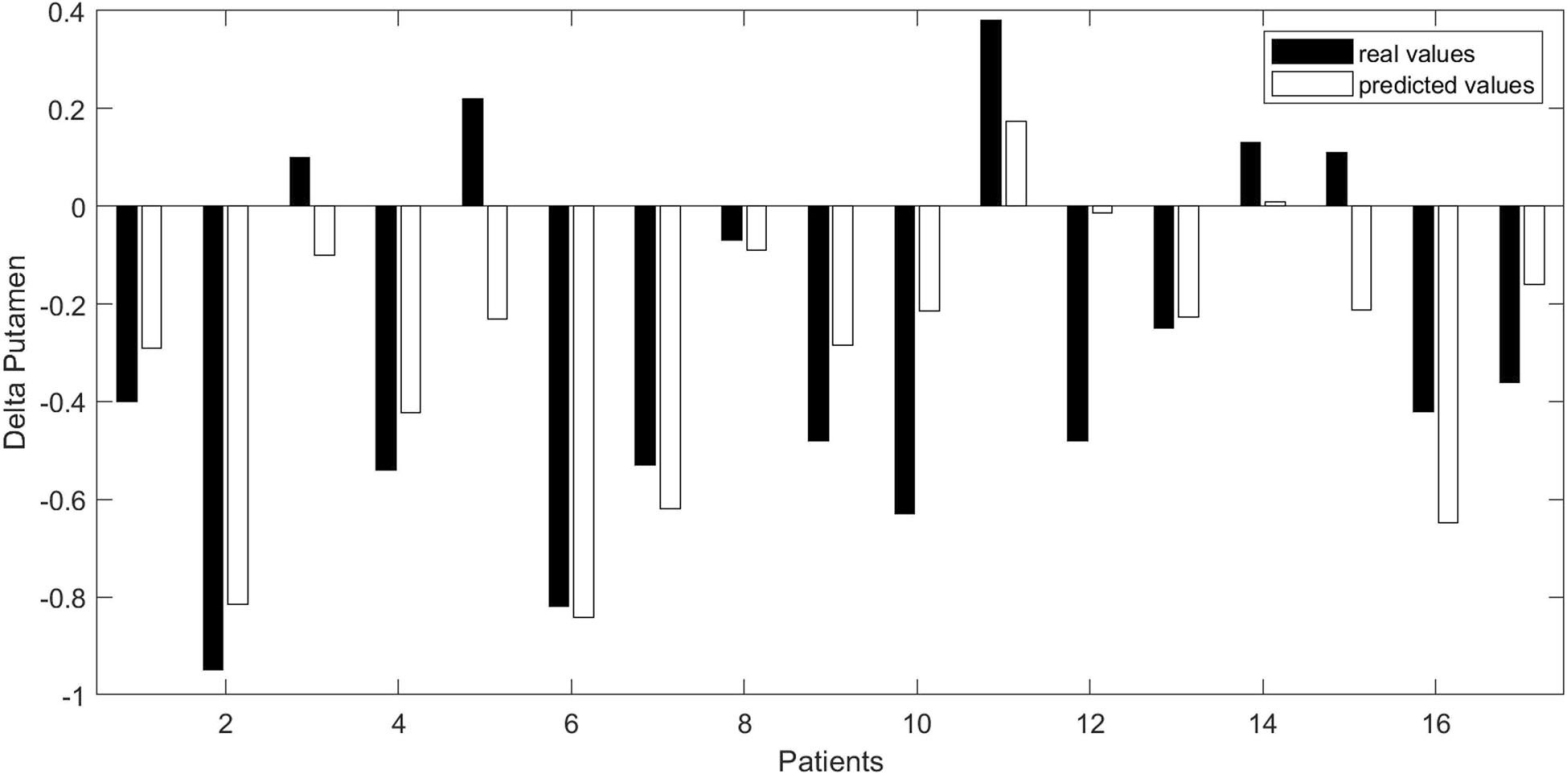
Linear regression model predicting the decreased activity of the putamen by *Δcortisol + ΔIL-6 + Δdopamine + constant* for the 17 patients

The high predictive power of the model (*F* (3,13) = 5.88, *p* = .009, R^2^ = .58, Table 4) is shown in Fig. 3. The result of the stepwise regression model including also non-significant variables is given in Table S3 (Supplement D). The robust version, which is less prone to outliers, did not alter the result (Supplement D).

**Table 4:** Result of the linear model explaining the decreased activity of the putamen by the change in cortisol, interleukin (IL) 6 and dopamine (R^2^ = .58)

Variable	β	SE	*T*	*p*
constant	.2021	.0754	2.68	.02
cortisol	.0002	.0001	2.56	.02
IL-6	.0166	.0060	2.79	.02
dopamine	.0024	.0008	2.95	.01

*β* (unstandardized) regression coefficients, *SE* standard error

No model was able to explain the other changes of neural activity (Δ ACC, OFC, PFC, and precuneus, separately used as dependent variables) when testing with the Δ neurochemical parameters as predictors.

None of the models aiming to predict the Δ symptom severities of the psychological level was significant.

## Discussion

### Pre/post treatment changes

The first part of the paper focuses on assessing changes induced by psychotherapy at the psychological, neural, and neurochemical levels separately. For the neural level, a literature research was done to identify regions of interest in the brain, i.e., regions that had changed during psychotherapy of OCD. The activity of 6 of these 32 ROIs was significantly reduced after psychotherapy in our sample (Table 1): the prefrontal and orbitofrontal cortex, the precuneus, two ROIs within the ACC, and the putamen. The change in the ACC was even significant at the whole-brain level (Table 2). In sum, the changes in these regions underline the role of the cortico-striato-thalamic-cortical (CSTC) model of OCD extending to emotion-related regions and confirm the impact psychotherapy has on neural activity. Note, however, that the thalamus was not found in a meta-analysis by Thorsen et al. [14] nor by any of the studies assessing pre-post treatment changes in OCD. The proposed hyperactivity of the thalamus and its inclusion in the OCD brain network should be revisited.

Taking a closer look at the literature review (Table 1), the results seemed inconsistent at first with respect to the direction of change, i.e., if the therapeutic effect led to increased or decreased activity in the ROIs. These inconsistencies resolved when taking into account the fMRI stimulation paradigm: for symptom provocation, which mainly addresses the emotional aspects of OCD [14], the activity had decreased after psychotherapy in all regions but the amygdala. In the ACC, for example, the activity was *higher* for executive tasks after treatment, but *lower* for emotional tasks (symptom provocation). The observation suggests – rather than a general hyperactivity in patients – that the *recruitment* of the ACC for different tasks has changed during psychotherapy.

At the neurochemical level, the changes were less clear. The means of the parameters had partly changed in the expected direction after treatment, i.e., an increase of the growth factor BDNF, serotonin, and dopamine, and a decrease in the immune parameter IL-6. For the immune parameters IL-10 and TNFα, and cortisol, however, the concentration was higher at the end of the therapy. This increase might be due to the stressful process of psychotherapy, especially for cortisol, which is known to be enhanced after continuously stressful situations [51]. The fact that none of the changes were significant is most possibly due to the huge variability of the data (see the high standard deviations in Table S2, Supplement C).

### Connecting the levels

The second part of the paper dealt with the aim to find a linkage between the psychological, neural, and neurochemical level that goes beyond correlations. As a first approximation, bilinear models (i.e., linear model with interaction terms) were assessed. The changed activity of the putamen is related to the sum of cortisol, IL-6, and dopamine. The putamen, part of the striatum, is one of the key regions in the cortico-striato-thalamic-cortical circuit of OCD [1, 8, 9, 52]. The decreased activity after psychotherapy in a symptom-provoking paradigm goes in line with the findings of Nakao et al. [19]. This decrease is related in our model to an increase of the concentration of dopamine. The link between dopamine and the neural activity of the putamen is not surprising, given that it is the predominant neurotransmitter in this brain region. Of course, the concentration of dopamine in the serum cannot be equalized with its concentration within the putamen or in the cerebrospinal fluid, but lower levels of dopamine compared to controls have been reported in the serum of OCD patients (although with comorbid epilepsy) [53]. Moreover, subgroups of OCD patients show stereotypical motor behavior comparable to Parkinson patients – a disease that shares the impaired basal ganglia circuits with OCD and that is known for reduced levels of dopamine. Although the role of dopamine in OCD is not fully understood yet, and some studies reported elevated levels in OCD patients [25, 54], our results suggest that an increase of dopamine reduces the activity of the putamen, which again is associated with successful psychotherapy of the patients. A possible explanation for the contradicting results concerning the level of dopamine is provided by Belujon & Grace [55], who showed that acute stress is associate with an increase of dopamine concentration, followed by a decrease.

Next to dopamine, the activity of the putamen is influenced by interleukin 6 (IL-6) in our model. Cytokines like IL-6 influence the biochemical mechanisms of other cells, e.g., their inflammatory responses [56] and the concentration of neurotransmitters [57, 58], thus might have an additional indirect impact on psychiatric disorders. But also effects in the other direction are possible, since dopamine has been shown to increase the level of IL-6 [59].

The effect of cortisol on OCD is most likely due to its involvement in the stress responses [55]. The concentration of cortisol is known to be elevated in continuously stressful situations, which is proposed to be one of the reasons for the executive dysfunction and cognitive inflexibility commonly reported in OCD patients [51]. At first glance, our results contradict the consistent finding that cortisol is enhanced in OCD patients compared to controls [60–63]. However, a successful psychotherapy is a stressful period in life, especially when the patient is willing to work on his problems. Enhanced levels of cortisol could therefore be interpreted as an indicator of intense therapeutic work of the patient, which then leads to decreased activity in the putamen.

Notably, no reliable relation was found between the psychological level and the other levels, although the missing relation to the OCD symptom severity (Y-BOCS) is in accord with the literature: few studies report significant results at all, and those are not convergent (supplement B). It remains an open question why the OCD symptom scores, in contrast to depressive symptom ratings, are not (at least not reliably) connectable to the neural level.

### Limitations and future research

Surely, a larger sample size would have been desirable to increase power and reduce the risk of false-negative findings. Nevertheless, we argue that (1) large effects, i.e., those of particular interest, are detectable with relatively small sample sizes, and (2) that power also depends upon sufficient individual-level data, e.g., by scanning for 20 instead of 10 min [64], as it was done here.

One limitation is that controls did not undergo venipunctures, so no comparison to controls was possible for the neurochemical parameters.

Note that in line with the aim of the project (identifying changes during treatment, not assessing the effectiveness of a certain psychotherapeutic approach), we do not consider the patients’ comorbidities and medication or the naturalistic (non-manualized) therapeutic setting as a limitation.

In future research projects, several aspects should be taken into consideration. First, neither the choice of brain regions (ROIs) nor the choice of neurochemical parameters were exhaustive in this study. For example, one could also test glutamate, which is known to be important for cognitive flexibility [65], or norepinephrine, which is involved in response inhibition [51]. Also, other parameters than the concentration might be relevant for psychiatric disorders, e.g., the functioning of receptors, firing, or synthesis rates.

Second, improvements should be made on the conceptual level by using more refined (nonlinear) models that include feedback loops, since only such models are able to produce self-organized behavior [66]. Last but not least, pre/post treatment analyses should be extended to assess additional time points within the therapy process in order to account for the dynamic nature of the psychotherapy process [67].

## Conclusion

After 2–3 months of inpatient psychotherapy, the symptoms of patients with obsessive-compulsive disorder had significantly decreased. On the neural level, significant reductions in the abnormal hyperactivity of brain regions of the cortico-striato-thalamo-cortical circuit were observed. On the neurochemical level, the changes were less clear due to the high variability of the parameter values. While no model was able to explain the changes in symptom severities, a highly significant and relevant regression model (R^2^ = .58) was found that explained the decreased neural activity of the putamen by increases of the concentrations of dopamine, the immune factor IL-6, and cortisol. Reduced activity of the putamen is usually associated with improvements of OCD patients, as shown in our study by the reduced activity during psychotherapy, although it was not directly related to the OCD symptoms measured by the Y-BOCS. More research is needed to gain an understanding of the complex interactions between the different levels of psychiatric disorders.

## Supplementary information

Additional file 1. In Supplementary_Material.pdf we provide details on the fMRI data acquisition and processing (Supplement A), the coordinates of all ROIs that were tested (Supplement B), the results of the neurochemical parameters that were not significant (Supplement C), and additional information on the stepwise and robust versions of the linear regression model (Supplement D). (DOCX 764 kb)

## Data Availability

The datasets used and analyzed during the current study are available from the corresponding author on reasonable request.

## Abbreviations

Δ: Pre-post difference
ACC: Anterior cingulate cortex
BDI-II: Beck Depression Inventory II
BDNF: Brain-derived neurotrophic factor
CSTS: Cortico-striato-thalamic-cortical circuit
dlPFC: Dorso-lateral PFC
FDR: False discovery rate
fMRI: Functional magnetic resonance imaging
FWE: Family-wise error
IL-6: Interleukin 6
IL-10: Interleukin 10
OCD: Obsessive-compulsive disorder
OFC: Orbito-frontal cortex
PCu: Precuneus
PFC: Prefrontal cortex
ROI: Region of interest
SCL-90-R: Symptom Scales 90 revised
SD: Standard deviation
TNF-α: Tumor necrosis factor alpha
vlPFC: Ventro-lateral PFC
Y-BOCS: Yale-Brown Obsessive Compulsive Score

## Footnotes

1 http://www.click2go.umip.com/i/software/mrm.html

2 http://marsbar.sourceforge.net/

3 http://www.inside-r.org/r-doc/stats/p.adjust
