# Supplementary material for: Multi-level assessment of obsessive-compulsive disorder (OCD) reveals relations between neural and neurochemical levels
Source: BMC Psychiatry. 2020 Nov 25;20:559. doi: 10.1186/s12888-020-02913-5 (PMC7687999; doi:10.1186/s12888-020-02913-5)
Supplement: Supplementary file 1 — In Supplementary_Material.pdf we provide details on the fMRI data acquisition and processing (Supplement A), the coordinates of all ROIs that were tested (Supplement B), the results of the neurochemical parameters that were not significant (Supplement C), and additional information on the stepwise and robust versions of the linear regression model (Supplement D). (DOCX 764 kb) [file 12888_2020_2913_MOESM1_ESM.pdf]

# Supplement A: fMRI data acquisition and processing

**Data acquisition**

Images were acquired with a 3T Siemens TIM TRIO whole-body scanner (Siemens Symphony, Erlangen, Germany) with a 32-channel head coil. First, a high-resolution scan was acquired for anatomical referencing using a T1-weighted MPRAGE sequence (FoV: 256 mm, slice thickness 1.0 mm, TR 2300 ms, flip angle 9°, resolution:1x1x1 mm). Functional images were obtained in two sessions with a short pause in between. Concerning the functional imaging, a total of 552 volumes were acquired using a T2*-weighted gradient echo EPI with 36 slices (slice thickness=3 mm, descending slice order, TR=2.25 s, TE=30 ms, flip angle =70°, FoV=192 mm). The first 6 volumes of each functional session were discarded due to saturation effects, leaving a total of 540 volumes. Stimuli were presented with the E-Prime 2.0 presentation software[1] as an event-related design in a pseudo-randomized order in 2 sessions (20 pictures of each category in each session). The pictures were shown for 4 seconds each, separated by a fixation cross; the inter-stimulus interval was 2 seconds. The resulting DICOM files were converted to 4D-NIfTI-files with the tools MRIConvert[2] and dcm2nii[3].

**Preprocessing**

Preprocessing and statistical analyses were performed using the Statistical Parametric Mapping software package SPM12 (Wellcome Department of Cognitive Neurology, London) implemented in Matlab (Mathworks, Inc., Natick, MA, USA, release 13a). Functional images were realigned to the first image, de-spiked with the AFNI 3d-despike function[4], unwarped, corrected for geometric distortions using the fieldmap of each participant, and slice time corrected.

The high-resolution structural T1-weighted image of each participant was processed and normalized with the CAT12 toolbox[5] using default settings. Each structural image was segmented into gray matter, white matter, and CSF, and denoised, then warped into MNI space by registration to the DARTEL template provided by the CAT12 toolbox via the high-dimensional DARTEL registration algorithm [1]. Based on these steps, a skull stripped version of each image in native space was created. To normalize functional images into MNI space, the functional images were coregistered to the skull stripped structural image and the parameters from the DARTEL registration were used to warp the

[1] http://www.pstnet.com
[2] https://lcni.uoregon.edu/downloads/mriconvert/mriconvert-and-mcverter
[3] http://www.mccauslandcenter.sc.edu/crnl/tools
[4] https://afni.nimh.nih.gov
[5] http://dbm.neuro.uni-jena.de/cat

functional images, which were resampled to 3 × 3 × 3 mm voxels and smoothed with a 6 mm FWHM Gaussian kernel. The quality of the preprocessing was checked using the tools BXH[6] and tsdiffana[7].

**Statistical Analysis**

Since SPM uses a mass-univariate approach, the effect of the conditions were modeled for each voxel with a general linear model [2]. The movement parameters gained from the realignment procedure during preprocessing were used as regressors. Corresponding to the 4 categories of pictures shown during scanning, 4 conditions were modeled (individual OCD, standard OCD, disgusting, and neutral pictures). First-level contrasts were calculated for each participant and each scan for individual OCD vs. neutral pictures.

## Supplement B: ROIs for the neural level

A search in PubMed[8] yielded 10 studies that had assessed the change in neural activity after psychotherapy (Table 1). The study by Schiepek et al. [3] could not be used for ROI analysis due to missing MNI coordinates of the regions, and the study by Nakao et al. [4] was excluded due to the very loose threshold of $p < .1$ uncorrected. When Talaraich-coordinates were given [e.g., 5]), they were transformed to MNI space with the WFU pickatlas tool[9].

Freyer et al. [6] found a negative correlation of the intensity of OCD symptoms with the activity of the pallidum, Huyser et al. [7] a positive correlation with the prefrontal cortex, and Verfaillie et al. [8] reported positive correlations with left putamen, left OFC, left DLPFC, and right insula. Also, the predictive value of correlations between pre-treatment neural activity and changes in symptom severity yielded diverging results: while Sanematsu et al. [9] found predictive power for the cerebellum and the superior temporal gyrus, Olatunji et al. [10] reported that activation in the prefrontal cortex, the temporal pole, and the amygdala, was associated with better treatment response.

[6] https://wiki.biac.duke.edu/biac:xmlheader
[7] http://imaging.mrc-cbu.cam.ac.uk/imaging/DataDiagnostics
[8] https://www.ncbi.nlm.nih.gov/pubmed
[9] https://www.nitrc.org/projects/wfu_pickatlas/

*Table S1: Group mean (SD) values of the neural activity and FDR-corrected p-values for the group x time interaction term of the repeated measures ANOVA.*

| Region | Reference | L/R | coordinates | | | *p (FDR)* |
|---|---|---|---|---|---|---|
| | | | x | y | z | |
| **frontal lobe** | | | | | | |
| prefrontal | ↑ Huyser et al. [11] | L | -15 | 3 | 54 | .43 |
| | | L | -33 | 33 | 45 | .63 |
| | | R | 30 | 30 | 45 | .63 |
| | | R | 51 | -6 | 54 | .63 |
| middle frontal | ↓ Nabeyama et al. [5] | L | -24 | 25 | 43 | .44 |
| premotor | ↑ Huyser et al. [11] | L | -39 | 3 | 63 | .83 |
| | ↑ Huyser et al. [11] | R | 36 | -18 | 57 | .56 |
| orbitofrontal | ↓ Nabeyama et al. [5] | R | 22 | 44 | -5 | .87 |
| | ↓ Baioui et al. [12] | L | -45 | 17 | -8 | .03* |
| | ↓ Baioui et al. [12] | L | -48 | 17 | -5 | .03* |
| | ↓ Morgiève et al. [13] | L | -24 | 30 | -22 | .96 |
| | | R | 20 | 24 | 20 | .87 |
| **parietal lobe** | | | | | | |
| precuneus | ↓ Nabeyama et al. [5] | L | -36 | -76 | 33 | .69 |
| | | R | 4 | -72 | 46 | .03* |
| supramarginal, posterior | ↓ Baioui et al. [12] | L | -54 | -28 | 28 | .96 |
| | | R | 69 | -25 | 25 | .41 |
| **temporal lobe** | | | | | | |
| supramarginal, anterior | ↓ Baioui et al. [12] | L | -51 | -49 | 10 | .66 |
| insula | ↓ Lázaro et al. [14] | L | -40 | -12 | -14 | .81 |
| | ↓ van der Straten et al. [15] | L | -36 | 26 | -5 | .44 |
| fusiform gyrus | ↓ Nabeyama et al. [5] | L | -47 | -30 | -26 | .71 |
| **limbic lobe** | | | | | | |
| anterior cingulate | ↑ Huyser et al. [11] | L | -9 | 21 | 42 | .03* |
| | ↓ Verfaillie et al. [8] | R | 6 | 35 | 6 | .43 |
| | ↓ Morgiève et al. [13] | L | -4 | 28 | 24 | .02* |
| parahippocampus | ↓ Nabeyama et al. [5] | L | -24 | -26 | -25 | .44 |
| | ↓ Nabeyama et al. [5] | R | 26 | -30 | -21 | .69 |
| **basal ganglia** | | | | | | |
| n. accumbens | ↓ Baioui et al. [12] | L | -6 | 14 | -5 | .59 |
| n. caudatus | ↑ Freyer et al. [6] | R | 6 | 6 | 9 | .63 |
| | ↓ Baioui et al. [12] | R | 18 | 2 | 22 | .87 |
| putamen | ↓ Lázaro et al. [14] | L | -32 | 2 | -16 | .44 |
| | ↑ Verfaillie et al. [8] | L | -15 | 2 | 1 | .03* |
| **cerebellum** | | | | | | |
| declive | ↑ Nabeyama et al. [5] | L | -26 | -83 | -29 | .87 |
| cerebellar tonsil | ↑ Nabeyama et al. [5] | R | 49 | -58 | -49 | .59 |

* significant at $p < .05$ (FDR-corrected). L/R: left/right hemisphere. Note that the table includes also studies without controls [8], and many without correction for multiple comparison (all but Baioui et al. [12] and van der Straten et al.[15]).

## Supplement C: Results of the neurochemical parameters

The changes of the neurochemical parameters, which were assessed from the serum of the patients taken at 8 a.m., did not show significantly alterations during the process of psychotherapy. The non-significance can probably be partly explained by the high variability of the parameters, as can be seen in the high standard deviations of Table S2.

*Table S2: Change in neurochemical variables of patients before and after psychotherapy with mean (SD) and p-values of the 2-sided paired t-test.*

| Variable | mean pre | mean post | *p* |
|---|---|---|---|
| Cortisol (pg/ml) | 274.37 (217.80) | 701.13 (968.59) | .07 |
| BDNF (pg/ml) | 13.04 (9.16) | 57.03 (172.71) | .30 |
| IL-6 (pg/ml) | 23.39 (12.75) | 22.60 (11.71) | .78 |
| IL-10 (pg/ml) | 4.96 (2.30) | 5.09 (3.13) | .86 |
| TNFα (pg/ml) | 12.94 (4.23) | 15.10 (9.30) | .32 |
| Dopamine (pg/ml) | 41.11 (31.64) | 48.87 (81.77) | .72 |
| Serotonin (ng/ml) | 5.81 (4.91) | 8.60 (12.62) | .33 |

BDNF: brain-derived neurotrophic factor, IL: interleukin, TNFα: tumor necrosis factor.

## Supplement D: Details on the linear regression model

### Results of the stepwise linear regressions

The result of the stepwise bilinear regression in MATLAB for the model explaining the change in neural activity of the putamen is given in Table S3. The model's statistical characteristics were $R^2 = .78$, $F(6,10)=5.93$, and $p = .007$, but some of the variables/interaction terms were not significantly different from zero (last column in Table S3). Only the significant variables were then entered into a conventional linear regression model.

*Table S3: Result of the stepwise linear regression with Δactivity putamen as dependent variable and all (also the non-significant) predictors. .*

| Variable | estimate | SE | *T* | *p* (FDR) |
|---|---|---|---|---|
| constant | .163 | .063 | 2.577 | .05 |
| cortisol | <.001 | <.001 | -2.924 | .04 |
| IL6 | -.019 | .006 | -3.212 | .03 |
| dopamine | -.002 | .001 | -3.409 | .03 |
| serotonin | -.010 | .005 | -2.046 | .09 |
| cortisol*IL6 | <.001 | <.001 | -1.400 | .19 |
| cortisol*dopamine | <.001 | <.001 | 1.970 | .09 |

## Robustness of the linear regression

Least-square regression models can be prone to outliers, especially with small sample sizes, and are vulnerable to the violation of model assumptions, e.g., an asymptotic distribution of errors, and correlations between predictor variables. We therefore provide a) an analysis of possible outliers based on the Cook's Distance, b) the distribution of errors, and c) a comparison between the original data, the predicted response of the least-square regression, and the predicted response of the robust regression. The robust version of the regression was performed in MATLAB with the option '*RobustOpts'* enabled for the function *fitlm* (MathWorks, 2019).

Note that none of the variables were correlated.

Two values were determined as a possible outlier (Figure S1-A). Even though the distribution of the residuals did not suggest a distortion of this outlier (Figure S1-B), the regression was repeated with a robust regression algorithm. The result (Figure S1-C) was hardly distinguishable from the original regression and still significant ($F(13,3) = 5.08$, $p = .02$). The goodness-of-fit was only slightly reduced ($R^2 = .54$ compared to $R^2 = .58$). All parameters remained significant (Table S4).

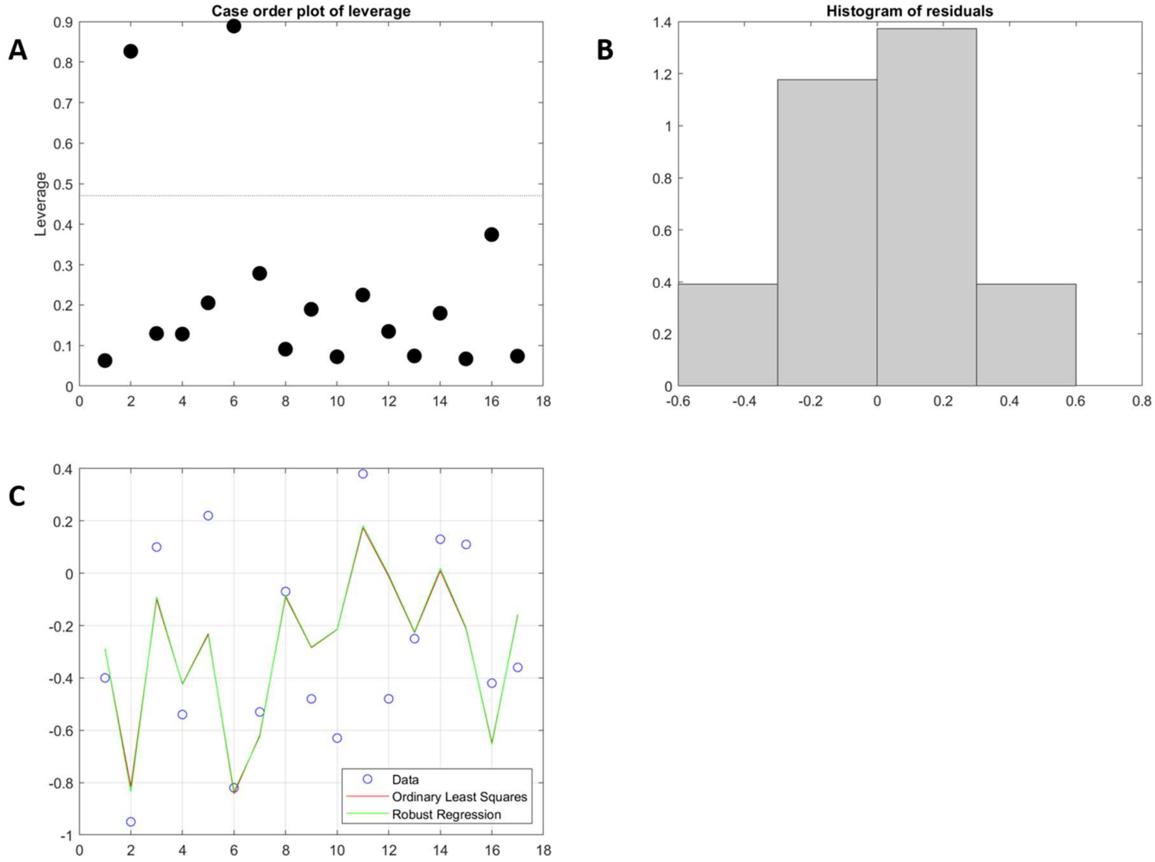


<u>Figure S1</u>: *Analysis of the data of model. A: Possible outliers (the dashed line corresponds to three times the mean of the Cook's Distance). B: Histogram of residuals. C: Comparison of the empirical data (blue dots), the result of the regression model with the normal algorithm (red), and with the robust version (green).*

*<u>Table S4:</u> Results of the normal ("original") and robust linear regressions predicting the Δ activity of the putamen.*

| | β | SE | *T* | *p* |
|---|---|---|---|---|
| Constant | | | | |
| original | -.202 | .075 | -2.679 | .019 |
| robust | -.198 | .082 | -2.399 | .032 |
| Cortisol | | | | |
| original | .000 | .000 | -2.559 | .024 |
| robust | .000 | .000 | -2.420 | .031 |
| IL6 | | | | |
| original | -.017 | .006 | -2.789 | .015 |
| robust | -.017 | .007 | -2.606 | .022 |
| Dopamine | | | | |
| original | -.002 | .001 | -2.949 | .011 |
| robust | -.002 | .001 | -2.692 | .018 |

## References

1. Ashburner J. A fast diffeomorphic image registration algorithm. Neuroimage. 2007;38:95–113. doi:10.1016/j.neuroimage.2007.07.007.

2. Kiebel SJ, Holmes AP. The general linear model. In: Statistical Parametric Mapping. 2007. p. 101–25.

3. Schiepek G, Tominschek I, Heinzel S, Aigner M, Dold M, Unger A, et al. Discontinuous Patterns of Brain Activation in the Psychotherapy Process of Obsessive-Compulsive Disorder: Converging Results from Repeated fMRI and Daily Self-Reports. PLoS One. 2013;8.

4. Nakao T, Nakagawa A, Yoshiura T, Nakatani E, Nabeyama M, Yoshizato C, et al. Brain activation of patients with obsessive-compulsive disorder during neuropsychological and symptom provocation tasks before and after symptom improvement: A functional magnetic resonance imaging study. Biol Psychiatry. 2005;57:901–10.

5. Nabeyama M, Nakagawa A, Yoshiura T, Nakao T, Nakatani E, Togao O, et al. Functional MRI study of brain activation alterations in patients with obsessive-compulsive disorder after symptom improvement. Psychiatry Res - Neuroimaging. 2008;163:236–47.

6. Freyer T, Klöppel S, Tüscher O, Kordon A, Zurowski B, Kuelz AK, et al. Frontostriatal activation in patients with obsessive-compulsive disorder before and after cognitive behavioral therapy. Psychol Med. 2011;41:207–16.

7. Huyser C, Veltman DJ, Wolters LH, De Haan E, Boer F. Functional magnetic resonance imaging during planning before and after cognitive-behavioral therapy in pediatric obsessive-compulsive disorder. J Am Acad Child Adolesc Psychiatry. 2010;49:1238-1248.e5. doi:10.1016/j.jaac.2010.08.007.

8. Verfaillie SCJ, de Wit SJ, Vriend C, Remijnse PL, Veltman DJ, van den Heuvel OA. The course of the neural correlates of reversal learning in obsessive-compulsive disorder and major depression: A naturalistic follow-up fMRI study. J Obsessive Compuls Relat Disord. 2016;9:51–8. doi:10.1016/j.jocrd.2016.02.004.

9. Sanematsu H, Nakao T, Yoshiura T, Nabeyama M, Togao O, Tomita M, et al. Predictors of treatment response to fluvoxamine in obsessive-compulsive disorder: An fMRI study. J Psychiatr Res. 2010;44:193–200. doi:10.1016/j.jpsychires.2009.08.007.

10. Olatunji BO, Ferreira-Garcia R, Caseras X, Fullana MA, Wooderson S, Speckens A, et al. Predicting response to cognitive behavioral therapy in contamination-based obsessive-compulsive disorder from functional magnetic resonance imaging. Psychol Med. 2014;44:2125–37.

11. Huyser C, Veltman DJ, Wolters LH, De Haan E, Boer F. Developmental aspects of error and high-conflict-related brain activity in pediatric obsessive-compulsive disorder: A fMRI study with a Flanker task before and after CBT. J Child Psychol Psychiatry Allied Discip. 2011;52:1251–60.

12. Baioui A, Pilgramm J, Kagerer S, Walter B, Vaitl D, Stark R. Neural correlates of symptom reduction after CBT in obsessive-compulsive washers-An fMRI symptom provocation study. J Obsessive Compuls Relat Disord. 2013;2:322–30. doi:10.1016/j.jocrd.2013.04.006.

13. Morgiève M, N'diaye K, Haynes WIA, Granger B, Clair AH, Pelissolo A, et al. Dynamics of psychotherapy-related cerebral haemodynamic changes in obsessive compulsive disorder using a personalized exposure task in functional magnetic resonance imaging. Psychol Med. 2014;44:1461–73.

14. Lázaro L, Caldú X, Junqué C, Bargalló N, Andrés S, Morer A, et al. Cerebral activation in children and adolescents with obsessive-compulsive disorder before and after treatment: A functional MRI study. J Psychiatr Res. 2008;42:1051–9.

15. van der Straten A, Huyser C, Wolters L, Denys D, van Wingen G. Long-Term Effects of Cognitive Behavioral Therapy on Planning and Prefrontal Cortex Function in Pediatric Obsessive-Compulsive Disorder. Biol Psychiatry Cogn Neurosci Neuroimaging. 2018;3:320–8. doi:10.1016/j.bpsc.2017.11.009.
